# Estimation of Early Graft Function Using the BETA-2 Score Following Clinical Islet Transplantation

**DOI:** 10.3389/ti.2022.10335

**Published:** 2022-07-06

**Authors:** Anna Lam, Richard A. Oram, Shareen Forbes, Tolu Olateju, Andrew J. Malcolm, Sharleen Imes, A. M. James Shapiro, Peter A. Senior

**Affiliations:** ^1^ Clinical Islet Transplant Program, Department of Medicine, University of Alberta and Alberta Health Services, Edmonton, AB, Canada; ^2^ Institute of Biomedical and Clinical Science, University of Exeter Medical School, University of Exeter, Exeter, United Kingdom; ^3^ BHF Centre for Cardiovascular Science, University of Edinburgh, Edinburgh, United Kingdom

**Keywords:** islet transplantation, graft survival, graft function, engraftment, BETA-2 score

## Abstract

Little is known about how early islet graft function evolves in the clinical setting. The BETA-2 score is a validated index of islet function that can be calculated from a single blood sample and lends itself to frequent monitoring of graft function. In this study, we characterized early graft function by calculating weekly BETA-2 score in recipients who achieved insulin independence after single transplant (group 1, *n* = 8) compared to recipients who required a second transplant before achieving insulin independence (group 2, *n* = 7). We also determined whether graft function 1-week post-transplant was associated with insulin independence in individuals who received initial transplant between 2000–2017 (*n* = 125). Our results show that graft function increased rapidly reaching a plateau 4–6 weeks post-transplant. The BETA-2 score was higher in group 1 compared to group 2 as early as 1-week post-transplant (15 + 3 vs. 9 + 2, *p* = 0.001). In an unselected cohort, BETA-2 at 1-week post-transplant was associated with graft survival as defined by insulin independence during median follow up of 12 months (range 2–119 months) with greater survival among those with BETA-2 score >10 (*p* < 0.001, log-rank test). These findings suggest that primary graft function is established within 4–6 weeks post-transplant and graft function at 1-week post-transplant predicts long-term transplant outcomes.

## Graphical Abstract

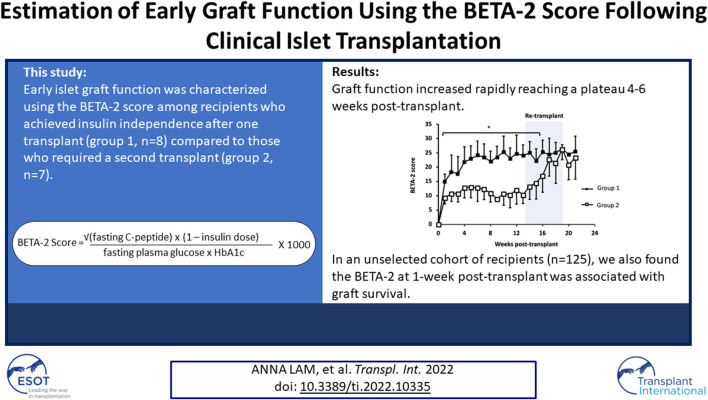

## Introduction

Advances in clinical islet transplantation including in islet processing and immunosuppression protocols have led to improved outcomes with increased rates of insulin independence and longer-lasting graft function (1). However, most recipients will require at least two islet transplants to achieve insulin independence and will have declining graft function over time with less than 50% of recipients maintaining insulin independence at 3 years post-transplant (2).

Optimization of early islet graft function remains an important target for improving long-term islet transplant outcomes. More than 50% of transplanted islets are lost in the first few days post-transplant (3, 4) and peri-transplant interventions limiting inflammation and islet stress have been shown to promote insulin independence and long-term islet survival (5, 6). Primary graft function at one-month post-transplant has been associated with long-term islet graft function (7), however, it remains unknown how primary graft function evolves in the first weeks to months after transplant.

One of the major challenges in this area has been the inability to closely monitor islet function. Formal stimulation tests measuring insulin or C-peptide response to stimuli such as glucose or arginine provide precise information on graft function, but the metabolic stress, as well as the time and labor-intensive nature of these tests, make them impractical for frequent monitoring in the clinical setting. Taking advantage of the BETA-2 score, a validated measure of islet function that can be calculated from a single fasting blood sample (8, 9), we characterized graft function in the first-weeks post-transplant and determined whether graft function as early as 1-week post-transplant is associated with long-term transplant outcomes.

## Methods

### Recipients

All subjects provided informed consent, and the analysis of data was approved by the University of Alberta Health Research Ethics Board. We performed a retrospective single-center analysis of individuals newly transplanted with allogeneic islets between 2009 and 2014. To characterize the establishment of islet graft function, BETA-2 score was calculated weekly in two selected groups representing distinct transplant outcomes: 1) subjects who achieved and maintained insulin independence for at least 12 months after a single islet infusion (group 1), and 2) subjects who only became insulin-independent (which was sustained beyond 12 months) after they received a second islet infusion after 3–6 months because they had not achieved insulin independence after their first infusion (group 2). Insulin independence was defined by no exogenous insulin use and no more than 2 self-monitored blood glucose levels >10.0 mmol/L during a 7-day period (10). A cohort of islet transplant recipients newly transplanted between 2000 and 2017 who had available lab results and insulin records at 1-week post-transplant were evaluated to determine whether BETA-2 score at 1-week post-transplant is associated with long-term transplant outcomes. The indications for islet transplantation, islet preparation, transplant procedure, and monitoring have been previously described (11, 12). Immunosuppression consisted of induction with alemtuzumab, thymoglobulin, daclizumab or basiliximab, and maintenance with tacrolimus and sirolimus or mycophenolate mofetil.

### Clinical Assessment

All subjects were seen weekly in-clinic during the first month post-transplant and then every 3–6 months in the first year post-transplant. Subjects were asked to self-monitor blood glucose and insulin usage. No specific protocol for insulin titration was used; post-transplant insulin doses were adjusted to avoid hyper- and hypo-glycemia (i.e., target glucose 4–10 mmol/L). Insulin dose (unit/kg) was calculated based on reported insulin dose divided by body weight measured at the most recent clinical assessment. Unfortunately, data on insulin delivery method was not available for this analysis. Blood work including fasting C-peptide and fasting glucose were measured every 1–2 weeks during the first 6 months post-transplant. HbA1c (as a percentage) was measured every 1–3 months post-transplant. For fasting blood work, patients were advised not to eat or drink after midnight the night before blood work was drawn with no specific instructions regarding insulin doses.

### Assays

Fasting plasma glucose concentrations were determined by the glucose oxidase method. C-peptide concentrations were measured using a commercial assay (Roche Elecsys; Roche Diagnostics, Indianapolis, IN). The lower limit of sensitivity for C-peptide in our laboratory was 0.02 nmol/L and the inter-assay coefficient of variation was 3.5%. HbA1c was measured by the Bio-Rad Variant II kit (Hercules, CA).

### Calculation of BETA-2 Score

BETA-2 scores were calculated weekly post-transplant. Derivation and validation of the BETA-2 score have previously been described (10). The BETA-2 is generated based on fasting C-peptide (nmol/L), daily insulin dose (units/kg), fasting plasma glucose (mmol/L), and HbA1c (%) as follows:

$$\text{BETA-}2\;\text{Score}=\frac{\sqrt{\left(\text{fasting}\;\text{C}‐\text{peptide}\right)}\text{ × }\left(1-\text{insulin}\;\text{dose}\right)}{\text{fasting}\;\text{plasma}\;\text{glucose}\;\text{ × }\;\text{HbA}1\text{c}}\text{ × }1000$$

### Other Indices of Islet Graft Function

Alternative simple indices of graft function were calculated at 1-week post-transplant as detailed below.

C-peptide/glucose ratio (CP/G) was calculated from C-peptide (ng/ml) and fasting plasma glucose (mg/dl) levels (13).

$$\text{CP}/\text{G}=\;\frac{\text{fasting C}‐\text{peptide }}{\text{ fasting plasma glucose}}\times 100$$

The homeostasis model assessment index of beta-cell function (HOMA2-B%) was calculated from fasting C-peptide (nmol/L) and plasma glucose (mmol/L) using the HOMA calculator (www.dtu.ox.ac.uk/homacalculator).

The Secretory Unit of Islet Transplant Objects (SUITO) index was also calculated from fasting plasma glucose (mmol/L) and C-peptide (nmol/L) (14, 15).

$$\text{SUITO}\;\;\text{index}=\frac{250\text{ × fasting}\;\text{C}‐\text{peptide}}{\text{fasting}\;\text{plasma}\;\text{glucose}-3\text{.}43}$$

Transplant estimated (TEF) was calculated from the daily insulin requirement (DIR; units/kg/24 h) and HbA1C (%) as previously described (16).

$$\text{TEF}=\left(\text{DIRpreTx}+\frac{\text{HbA}1\text{cpreTx}}{5\text{.}43}\right)‐\left(\text{DIR}+\frac{\text{HbA}1\text{c}}{5\text{.}43}\right)$$

### Statistics

Statistical analyses were performed using Stata version 14.1 (StataCorp, College Station, TX). Descriptive statistics are expressed as mean ± standard deviation (SD). Two-tailed t-test, Chi-square test, one-way ANOVA, and Tukey test were used to compare groups as appropriate. Receiver operating characteristic curves were constructed for recipients’ BETA-2 score, CP/G, HOMA2-B%, SUITO index, and TEF at 1-week post-transplant based on insulin independence. The association between BETA-2 score and insulin independence was evaluated by multiple logistic regression adjusted for pre-transplant BMI, HbA1C, and insulin dose, as well as islet equivalents per recipient body weight (IEQ/kg), transplanted. Survival analysis for the duration of insulin dependence was generated using the Kaplan-Meier method and analyzed using the Mantel-Cox log-rank test. A *p*-value < 0.05 was considered statistically significant and all *p*-values were reported as two-sided. To compare the differences in survival between groups, Bonferroni-adjusted posthoc pairwise comparisons were conducted with an adjusted *p*-value <0.017 considered statistically significant.

## Results

### Baseline Characteristics

The BETA-2 score was calculated on a weekly basis for the first 6 months post-transplant in 1) recipients who achieved insulin independence after a single transplant (*n* = 8, group 1) and 2) recipients who achieved insulin independence after having a second islet transplant 3–6 months from their first transplant (*n* = 7, group 2). Baseline characteristics were similar between both groups except for HbA1c which was higher in group 1 and BMI which was higher in group 2 (Table 1). Group 1 subjects received significantly higher islet equivalents per recipient body weight (IEQ/kg) with their first transplant compared to group 2 subjects (9476 ± 4205 IEQ/kg vs. 5603 ± 846 IEQ/kg, *p* = 0.03), however, there was no significant difference in total IE/kg after recipients from group 2 received their second transplant (9476 ± 4205 IEQ/kg vs. 13,094 ± 2711 IEQ/kg, *p* = 0.07).

**TABLE 1:** Baseline characteristics.

	All patients (*n* = 15)	Group 1 (*n* = 8)	Group 2 (*n* = 7)	*p*
Sex (male/female)	5/10	2/6	3/4	0.61
Age (years)	55.6 ± 9.9	56.8 + 9.4	54.3 ± 11.1	0.64
Diabetes duration (years)	34.9 ± 13.6	32.8 + 13.4	37.3 ± 14.4	0.54
Weight (kg)	68.5 ± 10.8	64.1 + 8.1	73.4 ± 11.9	0.10
BMI (kg/m^2^)	25.4 ± 2.6	23.9 + 1.9	27.0 ± 2.3	0.01
HbA1c (%)	8.6 ± 1.1	9.2 + 0.9	8.0 ± 0.9	0.03
Fasting blood glucose (mmol/L)	12.3 ± 5.4	13.5 + 4.9	11.0 ± 6.2	0.41
Insulin dose (units/kg per day)	0.5 ± 0.1	0.5 + 0.1	0.5 ± 0.1	0.96
First transplant				
IEQ	525,364 ± 274,102	624,189 + 348,429	412,422 ± 75,944	0.14
IEQ/kg	7,669 ± 3,626	9,476 + 4,205	5,603 ± 846	0.03
Second transplant				
IEQ			519,886 ± 176,138	
IEQ/kg			7491 ± 2,312	
Total IEQ	767,978 ± 328,107	624,189 + 348,429	932,308 ± 224,685	0.07
Total IEQ/kg	11,164 ± 3,935	9,476 + 4,205	13,094 ± 2711	0.07

BMI, body mass index; IEQ, islet equivalents; IEQ/kg, islet equivalents per recipient body weight. Data are expressed as mean ± SD and n (%).

### Early Graft Function

**FIGURE 1:**
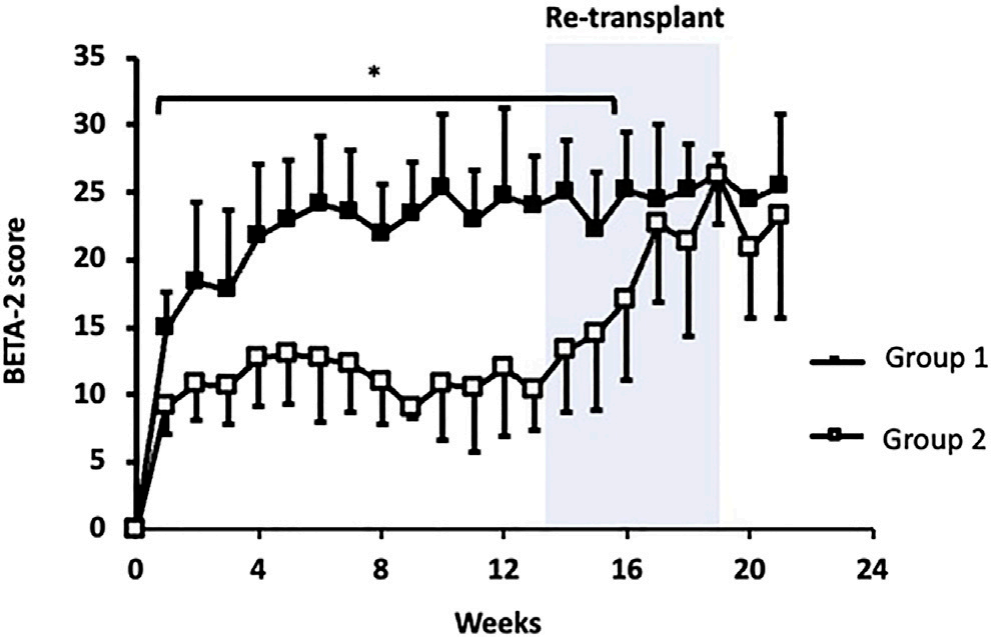
BETA-2 score in the first 6 months post initial islet transplant. Group 1 (closed squares). Group 2 (open squares). Shaded area indicates when group 2 received their second transplant. *p < 0.05, group 1 vs. group 2.

In both groups, BETA-2 score was measurable at 1-week and continued to increase before reaching a plateau 4 to 6 weeks post-transplant (Figure 1). BETA-2 score was significantly higher in group 1 compared to group 2 recipients as early as 1-week post-transplant (BETA-2 score 15 ± 3 vs. 9 ± 2, *p* = 0.001) and this difference was maintained until group 2 recipients received their second islet infusion at 4.1 ± 0.9 months (BETA-2 score 25 ± 4 vs. 17 ± 6, *p* = 0.07) (Figure 1). As expected, glycemic control as measured by HbA1c improved post-transplant in both groups (Supplementary Figure S1).

### Early Graft Function and Transplant Outcomes

**FIGURE 2:**
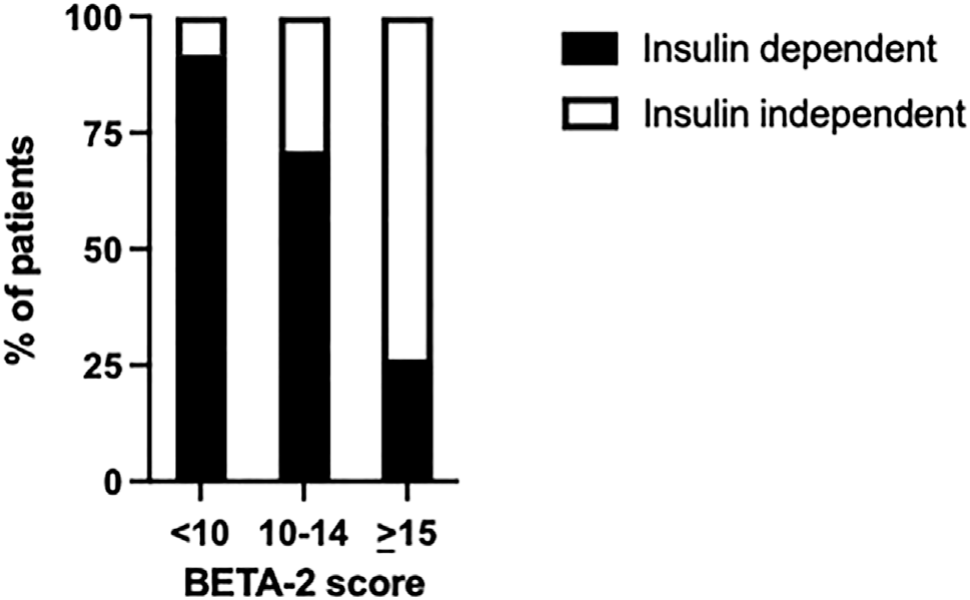
Percentage of transplant recipients who achieved insulin independence or remained insulin-dependent according to BETA-2 score at 1-week post-transplant *p* < 0.001.

**FIGURE 3:**
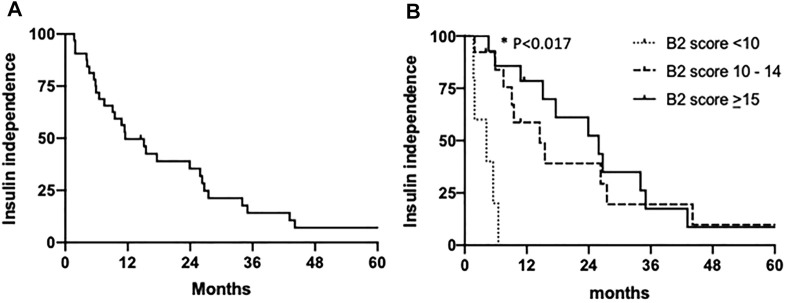
Kaplan-Meier estimates of the proportions of patients with insulin independence **(A)** among the entire cohort and **(B)** according to 1-week post-transplant BETA-2 score <10 (*n* = 5), 10–14 (*n* = 13) or >15 (*n* = 14). *Durability of insulin independence was significantly lower among subjects with 1-week BETA-2 score <10 vs. 10–14 (*p* = 0.0002) and BETA-2 score <10 vs. > 15 (*p* = 0.0001) by Mantel-Cox log-rank test and Bonferroni corrected significance threshold *p* < 0.017.

BETA-2 score at 1-week post-transplant was evaluated in an unselected cohort of recipients after their first islet transplant (*n* = 125) (Table 2). In total 26% achieved insulin independence for a median duration of 10 months (range 1.7–43 months, *n* = 32) while 74% remained insulin-dependent (*n* = 93). BETA-2 score at 1-week post-transplant was higher among those who achieved insulin independence compared to those who remained insulin-dependent (13 ± 3 vs. 9 ± 4, *p* < 0.001). BETA-2 score at 1-week also showed good discriminative ability for insulin independence (AUROC 0.83, *p* < 0.001) compared to alternative indices of graft function including the SUITO index, HOMA2-B%, CP/G and TEF (AUROC 0.55–0.77) (Supplementary Figure S2; Supplementary Table S1). Insulin independence was achieved in 8% (n = 5), 29% (*n* = 13), and 74% (*n* = 14) of recipients with BETA-2 score <10, 10–14 and ≥15, respectively (*p* < 0.001) (Figure 2). The odds of insulin independence increased with increasing BETA-2 score at 1 week including when adjusted for pre-transplant insulin dose, BMI, and HbA1c, as well as IE/kg transplanted (unadjusted odds ratio 1.39, 95% CI 1.21–1.59, *p* < 0.001 and adjusted odds ratio 1.44, 95% CI 1.23–1.70, *p* < 0.001). BETA-2 score at 1-week post-transplant was associated with graft survival as defined by insulin independence (*p* < 0.001, log-rank test) over a median follow-up of 12 months (range 2–119 months), with median survival of 4.2 months [IQR 1.9–5.5], 14.5 months [IQR 9.1–27.5] and 25.9 [IQR 15.1–35.0], respectively among recipients with BETA-2 score <10, 10–14 and ≥15 (BETA-2 score <10 vs. 10–14, *p* < 0.002 and vs. ≥15, *p* < 0.001) (Figure 3).

**TABLE 2:** Baseline characteristics of individuals newly transplanted between 2000–2017.

	All patients	BETA-2 score at 1-week post-transplant			*p*
		<10	10–14	≥15	
n	125	61	45	19	
Sex (male/female)	55/70	29/32	16/29	10/9	0.33
Age (years)	48.3 ± 9.8	46.5 ± 10.7	49.6 ± 8.7	51.0 ± 9.0	0.12
Diabetes duration (years)	32.7 ± 10.7	31.0 ± 10.2	33.2 ± 11.1	36.7 ± 10.7	0.12
Weight (kg)	74.0 ± 12.3	73.8 ± 13.4	73.5 ± 11.3	75.5 ± 11.5	0.84
BMI (kg/m^2^)	25.9 ± 3.4	25.9 ± 3.7	25.9 ± 3.1	26.2 ± 3.6	0.92
HbA1c (%)	8.3 ± 1.2	8.2 ± 1.3	8.3 ± 1.1	8.7 ± 1.3	0.25
Insulin dose (units/kg/day)	0.56 ± 0.16	0.60 ± 0.16	0.54 ± 0.16	0.50 ± 0.13	0.02
IEQ	465,565 ± 143,129	437,523 ± 147,628	483,862 ± 126,893	512,582 ± 152,524	0.08
IEQ/kg	6291 ± 1,581	5906 ± 1,482	6603 ± 1,532	6787 ± 1774	0.03

BMI, body mass index; IEQ, islet equivalents; IEQ/kg, islet equivalents per recipient body weight.

Data are expressed as mean ± SD and n (%).

## Conclusion

This study describes the evolution of islet graft function in the early period post-islet transplant using the BETA-2 score. This validated clinical score assessed weekly shows that graft function is established rapidly and increases over the first 4–6 weeks post-transplant before stabilizing. Furthermore, early engraftment estimated by the BETA-2 score as early as 1-week post-transplant is key to predicting longer-term transplant outcomes.

Vantyghem et al have shown that primary graft function as measured by the original BETA score at 1-month post-transplant is associated with prolonged graft survival (7). More recently, Witkowski et al demonstrated that the BETA-2 score on day 75 post-transplant is an early predictor of graft decline (15). In keeping with these studies, we found that it takes approximately 4–6 weeks before primary islet graft function is established and supports the association of graft function in the first 1–2 months with islet transplant outcomes.

Interestingly, our results suggest that it is possible to assess how well a graft will function even before primary graft function is fully established. We compared transplant recipients who achieved insulin independence for at least 1 year after a single transplant to those who remained insulin-dependent and found that the BETA-2 score was significantly higher at 1-week post-transplant among those who achieved insulin independence. We confirmed this in an unselected cohort of islet transplant recipients where a significantly higher BETA-2 score at 1-week was observed among those who achieved insulin independence post-transplant. In clinical practice, this may translate into earlier identification of recipients who are unlikely to achieve insulin independence and allow for earlier intervention including repeat transplantation in recipients who are already immunosuppressed/lymphodepleted. An early endpoint such as the BETA-2 score 1-week post-transplant could serve as an intermediate outcome and allow for shorter and more efficient clinical trial testing strategies designed to improve islet engraftment.

Ourselves and others have shown previously that BETA-2 scores >13 and >15 reliably predict insulin independence (8, 9) and a BETA-2 score >17.4 on day 75 post-islet transplant has been found to be associated with durable (5 years) insulin independence (15). This is similar to our current findings: that islet transplant recipients who achieved and maintained insulin independence for at least 1 year after a single infusion had an average BETA-2 score of 15 at 1-week post-transplant, and in our unselected cohort, recipients who achieved insulin independence (minimum duration 1 month) had average BETA-2 score of 13. In both analyses, for recipients who were unable to come off insulin, the average BETA-2 score at 1-week was 9. We also found that BETA-2 score at 1-week post-transplant was associated with long-term graft survival with a longer duration of insulin independence among recipients with BETA-2 scores of 10–14 and ≥15 compared to those with BETA-2 scores <10. Taken together, it appears that a BETA-2 score cut-off of >13 at 1-week post-transplant may be useful in identifying recipients who are likely to achieve insulin independence with higher scores being associated with a longer duration of insulin independence.

A potential limitation of the current analysis is the small number of subjects being compared in groups 1 (insulin-independent for > 1 year after a single transplant) and group 2 (recipients who did not become insulin-dependent until after a second transplant 3–6 months after the first infusion which was maintained at 12 months). This was necessary to be sure that the effect of each transplant could be assessed independently by selecting groups of recipients with distinct transplant outcomes, i.e., those with optimal vs. sub-optimal graft function. Thus, patients receiving a second transplant before 3 months were not included in case they might have been able to achieve insulin independence with the first transplant. Neither were recipients of second transplants who did not remain insulin independent at 12 months since the decline in graft function might be due to other factors such as rejection, rather than engraftment estimated by BETA-2. Most recipients at our center are re-listed for a second transplant at 4 weeks and priority is given to second infusions while recipients are still lymphodepleted. Furthermore, we confirmed that early graft function (1-week post-transplant) is associated with long-term transplant outcomes in an unselected cohort of transplant recipients with BETA-2 scores consistent with previous studies showing an association between BETA-2 scores and transplant outcomes (8, 9, 15).

A limitation of using the BETA-2 score soon after islet transplant is the inclusion of 1) HbA1c which is not expected to change in the short term and 2) insulin dose which may vary depending on several factors including diet, activity, and care provider discretion. However, in our study the BETA-2 score at 1-week post-transplant had better discrimination for insulin independence compared to other simple indices of islet function (SUITO index, HOMA2-B%, TEF and CP/G) suggesting that there is merit in including these additional variables even in short term assessment of graft function. Practical considerations for calculating the BETA-2 score peri-transplant may be to measure HbA1c less frequently (i.e., bi-weekly to monthly) than fasting C-peptide and glucose and to use standardized protocols regarding insulin dose adjustments.

Our study was not designed to explore how recipient and/or donor factors relate to graft function. However, we found that higher islet equivalents were associated with insulin independence and higher 1-week BETA-2 score in keeping with previous studies demonstrating single islet transplant success in recipients who had received higher transplanted islet mass(16, 17). Lower pre-transplant BMI and insulin requirements were also associated with higher BETA-2 scores at 1-week post-transplant suggesting that transplant success appears to depend not only on the number and function of transplanted islets but also on the metabolic demand placed on them. Importantly, however, we found that the association between insulin independence and BETA-2 score at 1-week post-transplant remained relatively unchanged when adjusted for pre-transplant BMI, insulin dose, and HbA1c, as well as transplanted IE/kg.

We characterized islet function in the early period post-transplant and show that primary graft function is established over the first 4–6 weeks post-transplant and that graft function as early as 1-week post-transplant is associated with long-term graft survival. Importantly, we demonstrated that frequent and close monitoring of islet graft function soon after transplantation is possible in the clinical setting and that this may be useful in routine clinical care as well as in the development and evaluation of interventions targeted at improving islet transplant outcomes.

## Data Availability

The data analyzed in this study is subject to the following licenses/restrictions: This study was a retrospectives analysis of single center data of individuals newly transplanted with allogenic islets between 2009–2014. The data is not publicly available. Requests to access these datasets should be directed to the corresponding author.

## Abbreviations

BMI, body mass index; CP/G, C-peptide/glucose ratio; HbA1c, hemoglobin A1c; HOMA2-B%, homeostasis model assessment index of beta cell function; IE, islet equivalents; SD, standard deviations; SUITO, Secretory Unit of Islet Transplant Objects; TEF, transplant estimated function.
