# Supplementary material for: Estimation of Early Graft Function Using the BETA-2 Score Following Clinical Islet Transplantation
Source: Transpl Int. 2022 Jul 6;35:10335. doi: 10.3389/ti.2022.10335 (PMC9301872; doi:10.3389/ti.2022.10335)
Supplement: Supplementary file 1 [file Table1.docx]

**Supplementary Table 1. Area under receiver operating curve (AUROC) of surrogate indices of graft function for the detection of insulin independence**

|  | **AUROC** | **95% CI** | **P** |
| --- | --- | --- | --- |
| BETA-2 | 0.83 | 0.75 – 0.91 | <0.001 |
| CP/G | 0.65 | 0.55 – 0.75 | 0.008 |
| HOMA2-B% | 0.77 | 0.68 – 0.86 | <0.001 |
| SUITO | 0.75 | 0.66 – 0.85 | <0.001 |
| TEF | 0.55 | 0.43 – 0.67 | 0.438 |
