# Supplementary figures and images for: Estimation of Early Graft Function Using the BETA-2 Score Following Clinical Islet Transplantation
Source: Transpl Int. 2022 Jul 6;35:10335. doi: 10.3389/ti.2022.10335 (PMC9301872; doi:10.3389/ti.2022.10335)

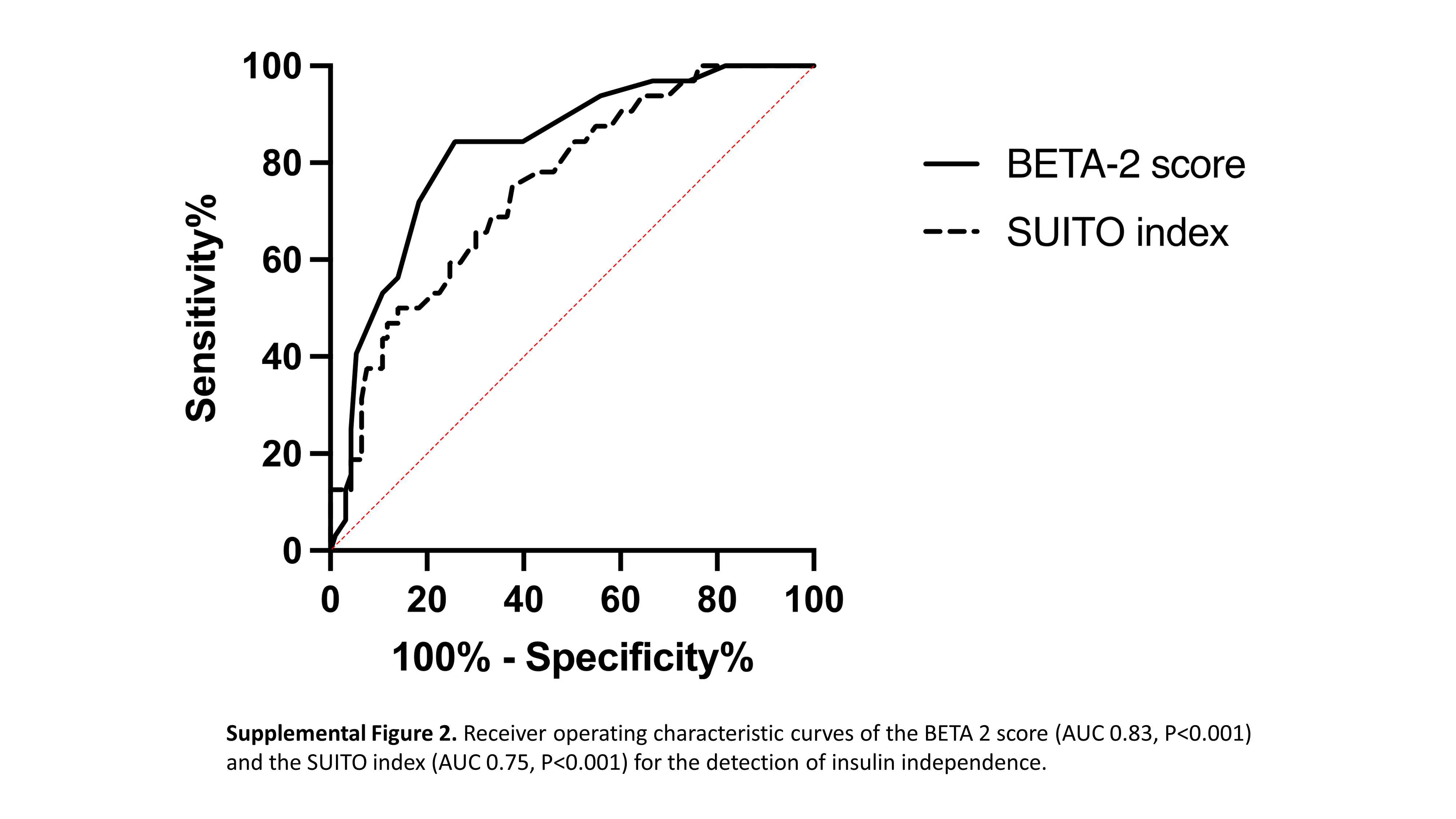

Supplement: Supplementary file 2 [file Image2.TIF]

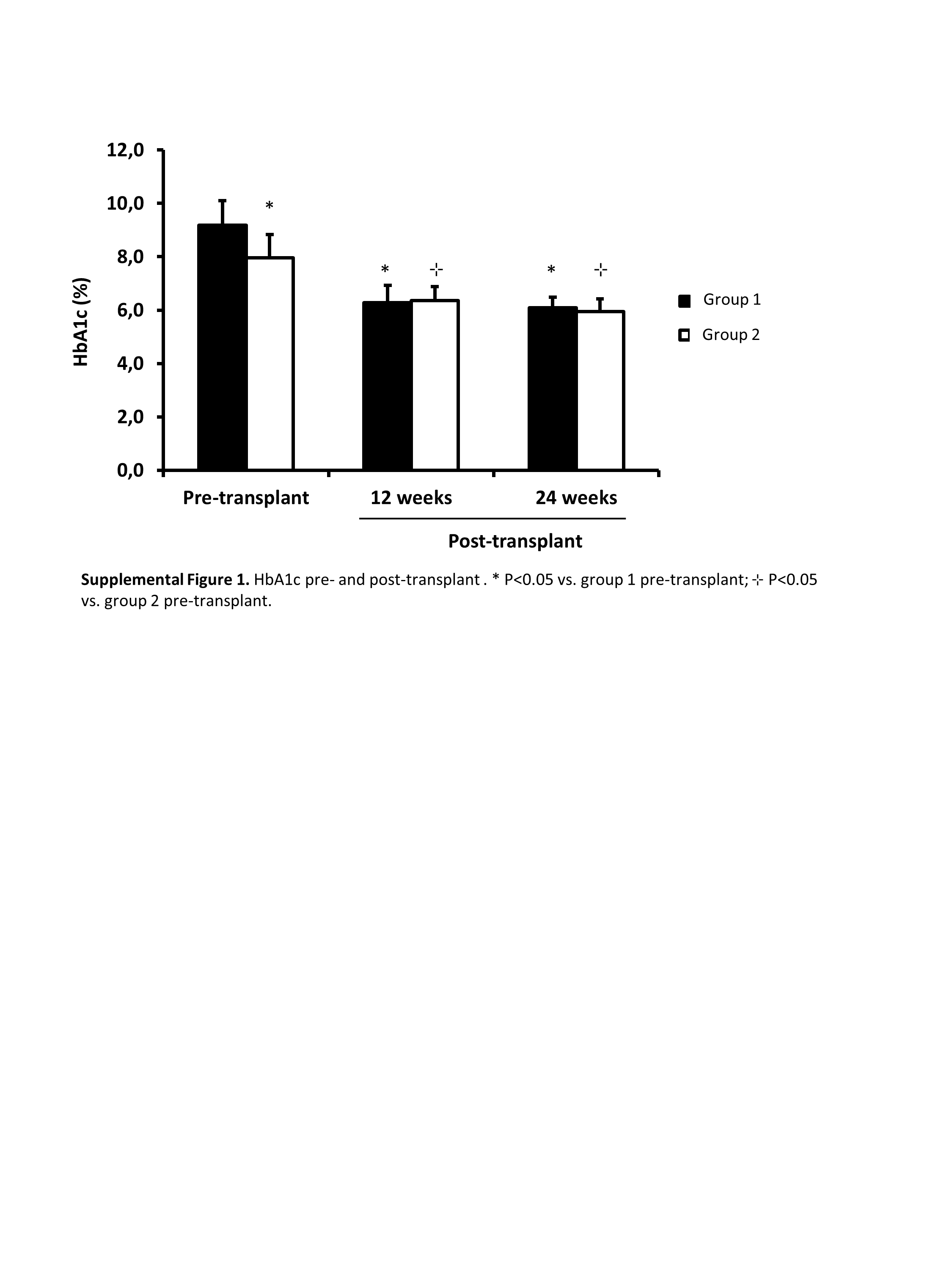

Supplement: Supplementary file 3 [file Image1.TIF]
